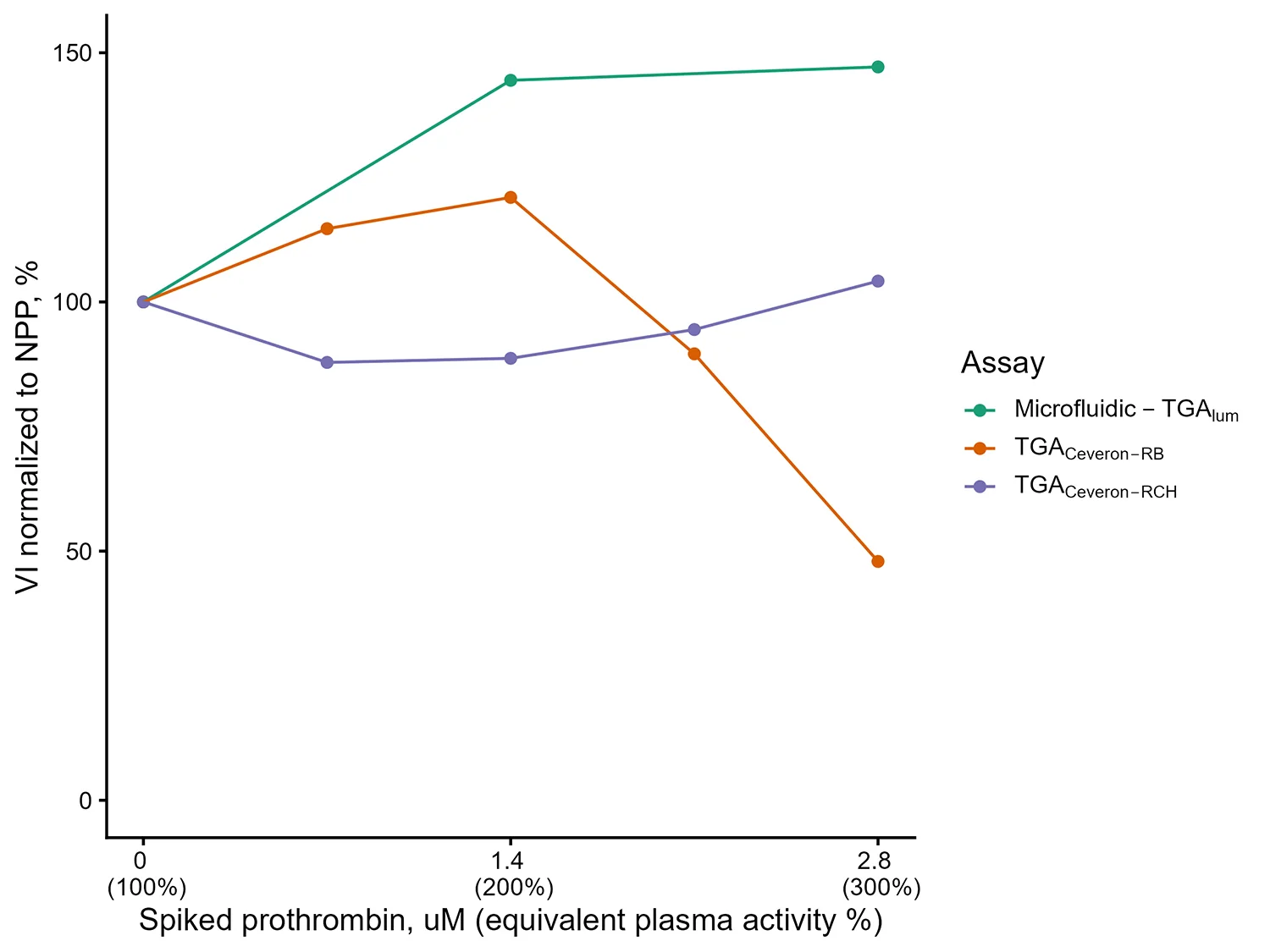# A miniaturized chemiluminescent thrombin generation assay to assess the effect of APCC in persons with hemophilia A with inhibitors: an observational study

**DOI:** 10.1016/j.rpth.2026.106920

**Published:** 2026-08-27

**Authors:** Aernoud P. Bavinck, Theodora J. Steeghs, Lisa van Engelshoven, Nerissa M.M. Coolen, Claudia J. van de Ridder, Beata Baran, Jerzy Windyga, Nicole M.A. Blijlevens, Saskia E.M. Schols, Cornelis van ’t Veer, Waander L. van Heerde

**Affiliations:** 1 Department of Hematology, Radboud University Medical Centre, The Netherlands; 2 Enzyre, Nijmegen, The Netherlands; 3 Laboratory of Hemostasis and Metabolic Diseases, Institute of Hematology and Transfusion Medicine, Warsaw, Poland; 4 Department of Hemostasis Disorders and Internal Medicine, Institute of Hematology and Transfusion Medicine, Warsaw, Poland; 5 Hemophilia Treatment Centre Nijmegen-Eindhoven-Maastricht, Nijmegen, The Netherlands; 6 Center for Infection and Molecular Medicine, Amsterdam UMC, Amsterdam, The Netherlands

**Keywords:** APCC, hemophilia A, microfluidic, blood coagulation tests

## Abstract

**Background:**

Monitoring of bypassing agents in persons with hemophilia A with inhibitors is complicated, especially when multiple treatment modalities are combined. The thrombin generation assay (TGA) might address these issues but is hampered by limited availability and standardization.

**Objectives:**

To assess the performance of a novel microfluidic chemiluminescent TGA (microfluidic-TGA_lum_) to monitor the effect of anti-inhibitor coagulant complex (APCC) in persons with hemophilia A with inhibitors.

**Methods:**

The microfluidic-TGA_lum_ was used in factor VIII (FVIII)-deficient plasma samples with different concentrations of FVIII inhibitors spiked with APCC and samples from 6 participants with hemophilia A with inhibitors before and after APCC administration. Peak height (PH), area under the curve (AUC), velocity index (VI), and lag time were compared with results obtained using the Ceveron TGA with reagent B (TGA_Ceveron-RB_).

**Results:**

Increasing concentrations of APCC spiked in FVIII-deficient plasma significantly increased PH, AUC, and VI and shortened lag time in the microfluidic-TGA_lum_, with strong correlation with TGA_Ceveron-RB_ outcomes (*r* = 0.92-0.97). Microfluidic-TGA_lum_ outcomes improved similarly after administering APCC to participants with hemophilia A with inhibitors, although thrombin generation exceeded normal values, with PH reaching 174% of that observed in normal pooled plasma. In contrast, the TGA_Ceveron-RB_ displayed persistently low thrombin generation and weak correlation with microfluidic-TGA_lum_ (*r* = 0.28-0.52). Exploratory follow-up experiments revealed that accumulation of prothrombin in plasma of participants with hemophilia A with inhibitors and low phospholipid concentration in the TGA_Ceveron-RB_ might explain this discrepancy.

**Conclusions:**

The microfluidic-TGA_lum_ detects the hemostatic effects of APCC treatment in persons with hemophilia A with inhibitors. Assay-specific differences in TGA response may complicate APCC monitoring in persons with hemophilia A with inhibitors. Our findings underscore the need for further mechanistic TGA studies and standardization.

## Essentials

• Activated prothrombin complex concentrate (APCC) is used in the treatment of hemophilia A.
• We assessed the ability of a novel microfluidic thrombin generation assay (TGA) to monitor APCC.
• The assay detected the effect of APCC in samples of persons with hemophilia A with inhibitors.
• The results differed from other TGAs, highlighting the need for standardization.

## 1 Introduction

Hemophilia A is a congenital X-linked disease, characterized by a decreased level of coagulation factor VIII (FVIII) activity. Traditionally, persons with hemophilia A receive FVIII concentrates to prevent and treat bleeding episodes [1,2]. However, an important complication of this treatment is the development of FVIII inhibitory antibodies, which occurs in approximately 30% of persons with severe hemophilia A [3,4].

In persons with hemophilia A with inhibitors who experience bleeding events, bypassing agents are administered to restore hemostasis [1]. Anti-inhibitor coagulant complex (APCC, FEIBA) has been used for this purpose for over 50 years [5]. APCC has been successfully used to treat bleeding in persons with hemophilia A with inhibitors [[6], [7], [8], [9]], as prophylaxis [8,10], and as periinterventional treatment [7,9]. It contains a mix of nonactivated and activated coagulation factors, with the activated factors being present at low levels (0.0002-0.004 U per U APCC) compared with their nonactivated counterparts (0.9-1.4 U per U APCC), except for FVIIa, which is present at higher levels (1.5-2.0 U per U APCC) [11]. FVII(a), FX(a) and prothrombin promote prothrombinase complex formation at the platelet surface, resulting in early thrombin generation if FV(a) is present [12,13]. The procoagulant effects of the other factor components, including FIX(a), are considered to be less critical, although they might contribute to the subsequent thrombin generation burst [11].

Recently, the nonfactor replacement therapy emicizumab has been introduced for long-term prophylaxis in persons with hemophilia A with or without inhibitors. Emicizumab functions by bridging FXIa and FX, effectively mimicking the role of FVIIIa in coagulation. Unfortunately, even with emicizumab, bleeding episodes still occur (breakthrough bleeds), which require concomitant therapy such as FVIII replacement therapy in persons with hemophilia A and bypassing agents in persons with hemophilia A with inhibitors [14,15].

Nonetheless, APCC use is not without limitations. Some persons with hemophilia A with inhibitors demonstrate reduced effect of APCC [16], but might be responsive to other bypassing agents, such as recombinant FVIIa [17]. Conversely, other persons with hemophilia A with inhibitors respond more favorably to APCC. While such between-patient differences in response indicate potential for personalized care, the choice of bypassing agents is currently primarily driven by the availability of products and the preferences of the treating team. Only if treatment fails, an alternative bypassing agent is initiated.

In addition to a lack of effect in some persons with hemophilia A with inhibitors, early reports of disseminated intravascular coagulation associated with APCC use raised concerns about its safety [[18], [19], [20]], although the incidence of thrombotic complications is ultimately low [7,21,22]. Discussions surrounding safety of APCC have rekindled after reports of thrombotic events in persons with hemophilia A with inhibitors treated with APCC in combination with emicizumab [23]. These reports led to a black box warning for the combined use of emicizumab and high doses (>100 U/kg/24 h) of APCC. However, more recent evidence suggests that the risk associated with combined use is less critical than originally believed [24]. Moreover, persons with hemophilia A with inhibitors treated with emicizumab who undergo surgery still have a high risk of bleeding despite concomitant treatment with bypassing agents, potentially indicating current undertreatment to avoid thrombotic complications [25].

Monitoring options are warranted both to enable selection of the optimal bypassing agent in persons with hemophilia A with inhibitors and to support monitoring and minimization of thrombotic risk [[26], [27], [28]]. The thrombin generation assay (TGA) has shown promise in this regard [16,29,30]. However, this assay is hampered by limited availability, validation, and clinical standardization [31], despite recent advances in this respect [32]. The turnaround time of current TGA platforms is long compared with that of other global hemostasis assays, such as viscoelastic assays, limiting its utility in emergency situations [33]. Additionally, TGA data on the effects of prolonged treatment with APCC are scarce.

The EnzySystem is a novel near-patient *in vitro* diagnostic platform aimed at measuring thrombin generation and FVIII activity levels in parallel directly in 1 sample within 1 hour. The development and technical validation of the FVIII activity assay [34] and TGA [35] performed in plasma were reported recently. This study evaluated the EnzySystem TGA for monitoring the effect of APCC in FVIII-deficient plasma and plasma retrieved from persons with hemophilia A with inhibitors and compared the results to a commercially available TGA platform.

## 2 Methods

### 2.1 Study outline

This study consisted of 2 parts. In the first part, commercially available FVIII-deficient plasma samples were spiked with increasing concentrations of APCC and tested with the microfluidic chemiluminescent TGA of the EnzySystem (microfluidic-TGA_lum_) and a commercially available fluorescent TGA with Ceveron reagent B (TGA_Ceveron-RB_) on the Ceveron S100 (Technoclone). In the second part, plasma samples obtained from persons with hemophilia A with inhibitors before and after routine prophylactic APCC administration were analyzed using the same TGAs.

Based on the results of the initial study components, several follow-up experiments were performed, including repeat testing of the samples with the Ceveron RC-High TGA kit (TGA_Ceveron-RCH_) and prothrombin spiking experiments across the different thrombin generation assays.

### 2.2 Patient and sample handling

Spiking experiments were performed with 3 commercially available FVIII-deficient citrated platelet-poor plasma samples (HRF Inc). One sample contained pooled plasma from 3 persons with severe hemophilia A without inhibitors. The other 2 samples were from persons with hemophilia A with inhibitors with inhibitor levels of 0.9 and 81 Nijmegen Bethesda units (NBU)/mL, respectively. The samples were spiked with 0, 0.25, 0.5, 1, and 2 U/mL APCC (FEIBA; Takeda), corresponding to *in vivo* doses of approximately 0, 15, 30, 60, and 125 U/kg [36].

The patient plasma samples used to study the effect of APCC were obtained from persons with hemophilia A with inhibitors who received treatment with APCC at the Institute of Hematology and Transfusion Medicine (IHT, Warsaw, Poland). Participants were identified and invited to participate in the study by their treating health care providers. Baseline characteristics were obtained with a short, structured interview in Polish. Citrated blood was drawn by regular venipuncture before and at 30, 120, and 240 minutes after a planned dose of APCC (50-70 U/kg bodyweight) by a trained phlebotomist at the IHT. The first tube collected of every draw was designated as a dummy tube and discarded. Immediately after venipuncture, samples were centrifuged twice at 1700*g* for 15 minutes to obtain platelet-poor plasma and samples were aliquoted. The TGA_Ceveron-RB_ was immediately performed on 1 aliquot of the sample. The remaining aliquots were snap frozen by submersion in a methanol dry-ice bath and stored at −80 °C until further testing at Enzyre’s laboratory (Nijmegen, The Netherlands). The microfluidic-TGA_lum_ was performed in duplicate on all samples. For all TGA measurements, only aliquots that had not been previously thawed were used, unless specifically stated otherwise.

### 2.3 Ethical considerations

This study adhered to the Declaration of Helsinki. The study protocol for the part of the study involving samples from persons with hemophilia A with inhibitors collected before and after APCC administration was approved by the ethical committee at the IHT (study A7-T4-2024) and registered on ClinicalTrials.gov (identifier NCT06357572). All participants provided written informed consent. The study is reported in accordance with the STROBE checklist for observational studies [37].

### 2.4 Assay methodology

**Figure 1:**
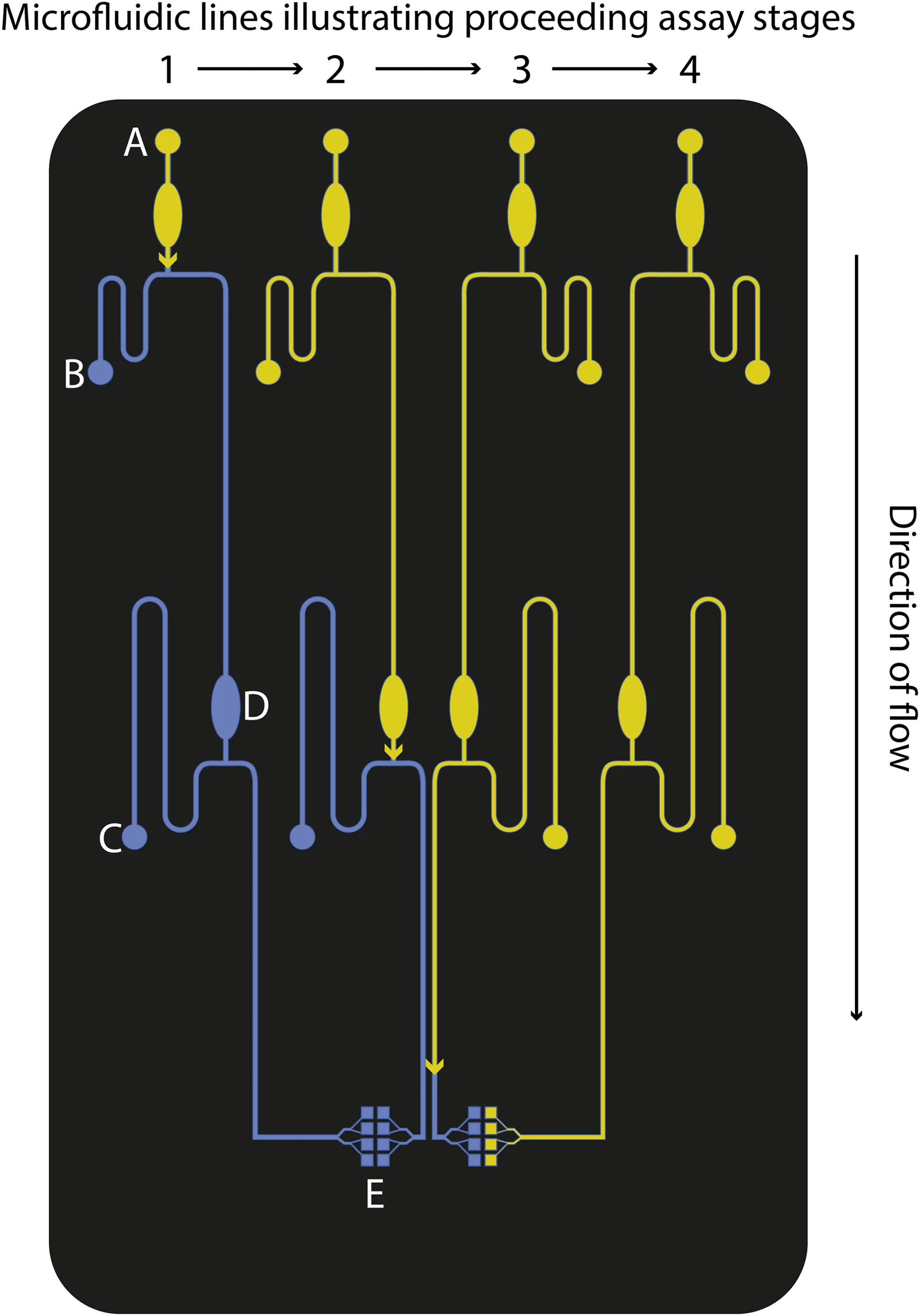
Overview of the EnzyCard The numbers (1-4) indicate the 4 individual microfluidic lines used to perform the thrombin generation assay. The plasma sample flow (yellow) is depicted across time, with line 1 representing the earliest state and line 4 the final stage of the assay. Letters (A–E) indicate specific structures in the microfluidic design: (A) Sample inlet, (B) First trigger line, (C) Second trigger line, (D) Second spotting chamber, (E) Detection chambers.

A detailed overview of the microfluidic-TGA_lum_ methodology was recently published [35]. In short, the microfluidic-TGA_lum_ was performed by pipetting 10 μL citrated plasma diluted 2:1 with buffer (0.5% bovine serum albumin, 25 mM HEPES, 125 mM NaCl, pH 7.4) into one of the sample inlets (Figure 1A). Each microfluidic card contains 4 independent microfluidic channels with individual sample inlets, allowing simultaneous measurement of 4 samples. Passive capillary force migrates the sample through the microfluidic channels. At specific points in the microfluidic geometry, stop valves prevent further microfluidic flow (Figure 1, line 1-2 arrowheads). At these points, microfluidic flow is resumed by manual pipetting of 10 μL of citrated plasma diluted 2:1 with buffer into the side channels, referred to as trigger lines (Figure 1B, C). The microfluidic design ensures that plasma inserted into the trigger lines mixes with the main sample at a 1:40 ratio. After reaching the second spotting chamber (Figure 1D), the trigger line is only activated after a 15-minute incubation period. This allows reconstitution and mixing of the sample with the dried-in reagents (final concentrations in parentheses) present in the spotting chamber: tissue factor (1 pM), phospholipids (16 μM), UltraGlo luciferase (20 μM). After the incubation period, the sample continues to flow through the microfluidic channels, ultimately entering 4 parallel detection chambers (Figure 1E). Each chamber contains the following dried-in reagents: CaCl_2_ (16 mM), β-AGR-aminoluciferin (1.1 mM), ATP (0.62 mM), MgCl_2_ (1.1 mM), and tissue-type plasminogen activator (0.6 μg/mL) (all final concentrations). Recalcification in the 37 °C thermostated detection chambers allows tissue factor-triggered activation of the coagulation cascade, resulting in the generation of thrombin. β-AGR-aminoluciferin is a substrate for thrombin. Upon cleavage of the β-AGR-aminoluciferin, free aminoluciferin is released, which is converted to oxyluciferin in the presence of luciferase, thereby releasing a photon. The generation of photons in each detection chamber is measured in real time and converted to relative light units (RLU) per second by an array of single-photon avalanche diode sensors positioned directly beneath the detection chambers. A USB cable provides the 12 volts for the sensor package and transfers the data to the computer for real-time display of the result and generation of a CSV dataset containing the signal over time.

For comparison, thrombin generation was measured with the TGA_Ceveron-RB_ on the Ceveron S100 according to the manufacturer’s instructions. This assay, which employs low levels of tissue factor and phospholipids, is intended to measure TGA in plasma samples of patients with bleeding disorders [38]. Based on the results of the TGA_Ceveron-RB_, the assay was repeated using the RC-High reagents (TGA_Ceveron-RCH_) on the same Ceveron S100 platform. This assay employs higher concentrations of tissue factor and phospholipids and is intended for monitoring anticoagulant treatment.

### 2.5 Data analysis

CSV files with measured data of microfluidic-TGA_lum_ were pseudonymously stored for analysis. For every microfluidic line, thrombin generation curves were obtained for the 4 individual detection chambers. The following TGA parameters were calculated for each detection chamber:

• Lag time: the time in seconds from the filling of the detection chamber thrombin generation starts.
• Peak height (PH): The highest signal measured during a run.
• Area under the curve (AUC): the area under the thrombin generation curve from the lag time until 1100 seconds thereafter.
• Velocity index (VI): The average slope of the thrombin generation curve from the time the signal reached 400 RLU until the peak height was attained.

Calculated parameters from the detection chambers were averaged to obtain a single value for each parameter, representing the outcomes of the assay.

To assess the effect of APCC treatment on TGA parameters, a repeated-measures ANOVA or a Friedman test was performed, depending on the normality of the data. In case of a significant effect, TGA parameters after APCC treatment were compared with pretreatment parameters using repeated-measures *t*-tests or Wilcoxon signed-rank tests with Holm correction for multiple testing. The results of the microfluidic-TGA_lum_ and TGA_Ceveron_ were compared with Pearson correlation.

Based on the findings from the comparison of TGA methodologies, an unplanned exploratory multilevel regression analysis was performed to assess the effect of between-patient variability on outcomes across both assays. In a multilevel regression, the intercept and/or the slope are permitted to vary between groups, here between persons with hemophilia A with inhibitors. These random effects, representing deviations of the parameter estimates from their baseline (fixed effect) values, are assumed to follow a normal distribution. By allowing such random variation, the model accounts for between-group differences. The model fit was compared between nested models with a chi-squared test and the Akaike Information Criterion (AIC); the latter corrects the improvement in fit for the added complexity of the model. Overall, a lower AIC indicates a better model fit.

All TGA parameters were calculated and all statistical analyses performed in R studio version 2024.04.2.

## 3 Results

### 3.1 Spiking effect of APCC in the microfluidic-TGA_lum_

**Figure 2:**
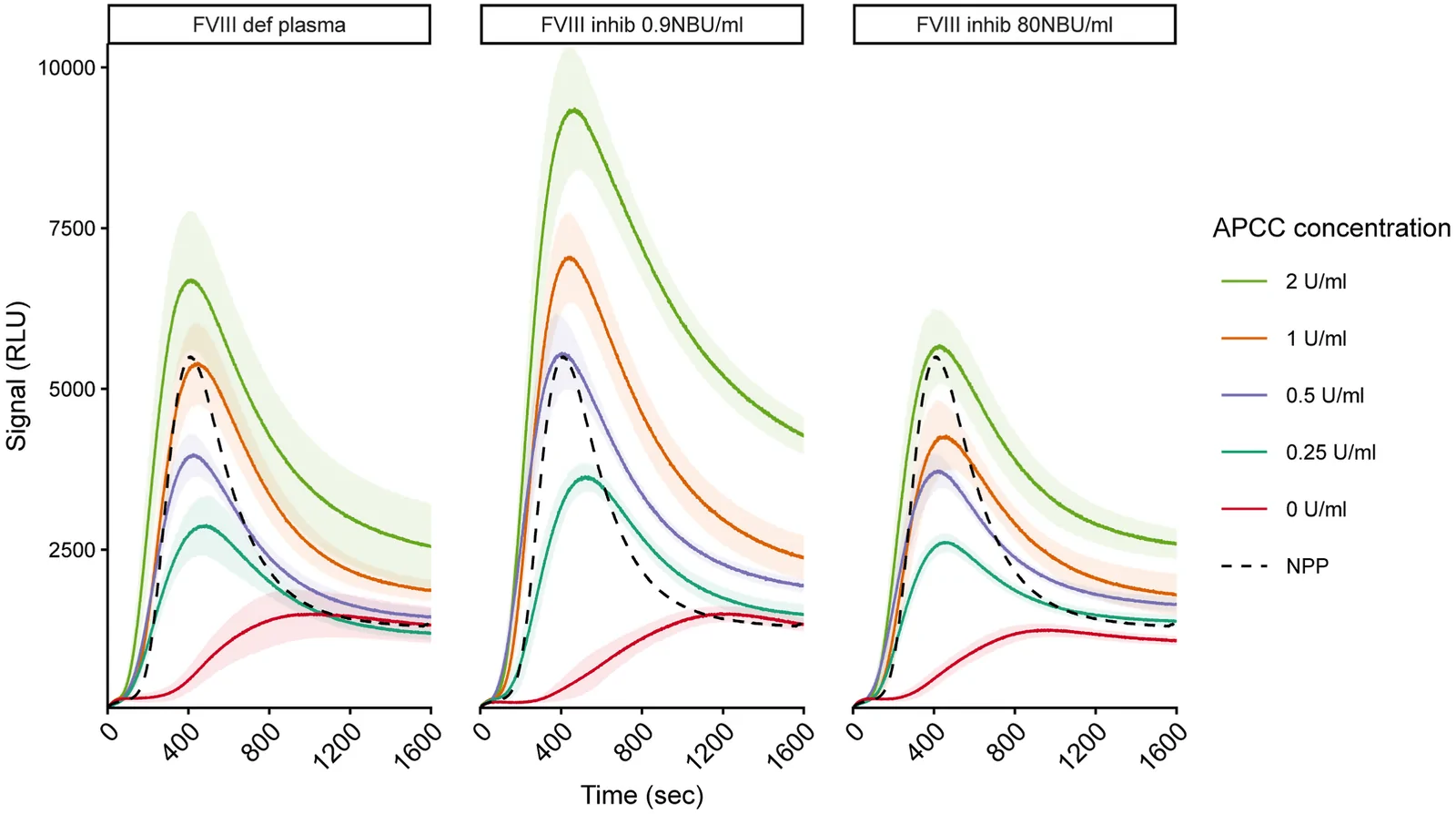
Microfluidic-TGA_lum_ curves in plasma samples spiked with APCC. Each curve represents the mean signal across 3 repeated measurements. Standard deviation is indicated by the colored area surrounding each curve. The dotted line represents the mean thrombin generation curve of 10 repeated measurements in NPP. APCC, anti-inhibitor coagulant complex; def, deficient; FVIII, factor VIII; inhib, inhibitors; NBU, Nijmegen Bethesda units; NPP, normal pooled plasma; TGA_lum_, chemiluminescent thrombin generation assay.

**Supplementary Figure S1:**
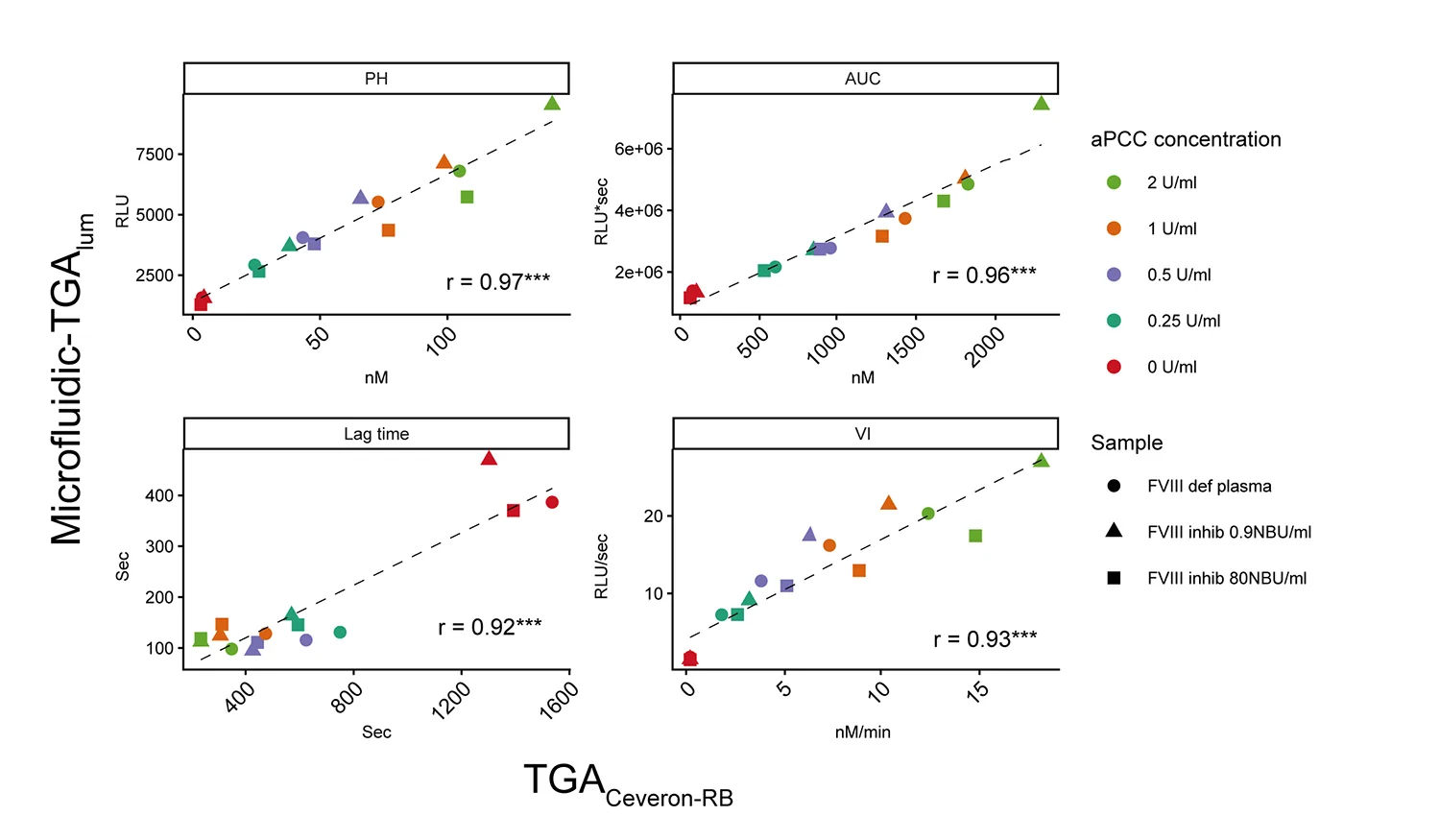


Increasing APCC concentrations in FVIII-deficient plasma samples dose-dependently enhanced thrombin generation in the microfluidic-TGA_lum_ (Figure 2). Without spiked APCC, thrombin generation was much lower in FVIII-deficient plasma than in normal pooled plasma (NPP; Figure 2, red line). The effects on PH, AUC, and VI increased in all samples up to the maximum concentration of 2 U/mL, whereas the maximum effect on lag time was reached at a concentration of 0.5 U/mL. The microfluidic-TGA_lum_ results correlated strongly with TGA_Ceveron-RB_ results, with Pearson correlation coefficients ranging from 0.92 to 0.97 across TGA parameters, with associated *P* values < .001 for all (Supplementary Figure 1).

### 3.2 Microfluidic-TGA_lum_ in samples of persons with hemophilia A with inhibitors treated with APCC

#### 3.2.1 Patient characteristics

Six persons with hemophilia A with inhibitors who were treated with APCC were included in the study. Baseline characteristics are reported in the Table. Two persons with hemophilia A with inhibitors (participants 2 and 5) received prophylactic treatment with emicizumab in addition to APCC. Participant 2 was prophylactically treated with APCC and emicizumab after experiencing significant bleeding during emicizumab monotherapy. Participant 5 did not receive regular APCC but received a single trial dose of APCC during this study in preparation for upcoming orthopedic surgery. Emicizumab-treated participants with hemophilia A with inhibitors received 50 U/kg APCC, compared to 70 U/kg received by other participants in this study. Annual bleeding rate was low in all participants with hemophilia A with inhibitors, except for participant 5, who experienced repeated bleeding episodes in a single target joint. None of the patients experienced thrombotic complications with the current treatment regime.

**Table:** Baseline characteristics.

Participants with hemophilia A	No. 1	No. 2	No. 3	No. 4	No. 5	No. 6
Age, y	65	54	41	78	50	59
Weight, kg	90	80	102	60	103	88
Ethnicity	European	European	European	European	European	European
Annual bleeding rate	1-3	0	0	0	5^a^	2^b^
Inhibitor level, NBU/mL	15.8	3.4	25.6	16.6	<0.5^c^	1.1
Prophylaxis	APCC 70 U/kg 2-3× per week	APCC 50 U/kg 2× per week	APCC 70 U/kg 3× per week	APCC 65 U/kg 3× per week	-d	APCC 70 U/kg 3× per week
		Emicizumab 3 mg/kg every 2 weeks			Emicizumab 1.5 mg/kg 1× per week	
Time after initiating APCC prophylaxis, y	7	1	1/3	7	NA	2
Time since last APCC, d	1	3	4	2	NA	2
APCC study dose, U/kg	70	50	70	70	50	70
Baseline prothrombin activity level, %	225	108	180	119	58	164
Prothrombin activity level after APCC treatment, %	524	295	>600^e^	319	287	447^f^

APCC, activated prothrombin complex concentrate; NA, not applicable; NBU, Nijmegen Bethesda units.

a All bleedings occurred in a single target joint.

b Hematuria in the presence of nephrolithiasis.

c History of inhibitor recurrence upon exposure to factor VIII concentrates and failed immune tolerance induction attempts.

d Patient received a trial dose of APCC in this study before upcoming orthopedic surgery.

e Prothrombin level was above the detectable range (150%) after 4 times dilution.

f Undiluted sample and the result exceeded the linear range, limiting the result’s reliability.

#### 3.2.2 Microfluidic-TGA_lum_ of persons with hemophilia A with inhibitors treated with APCC

**Figure 3:**
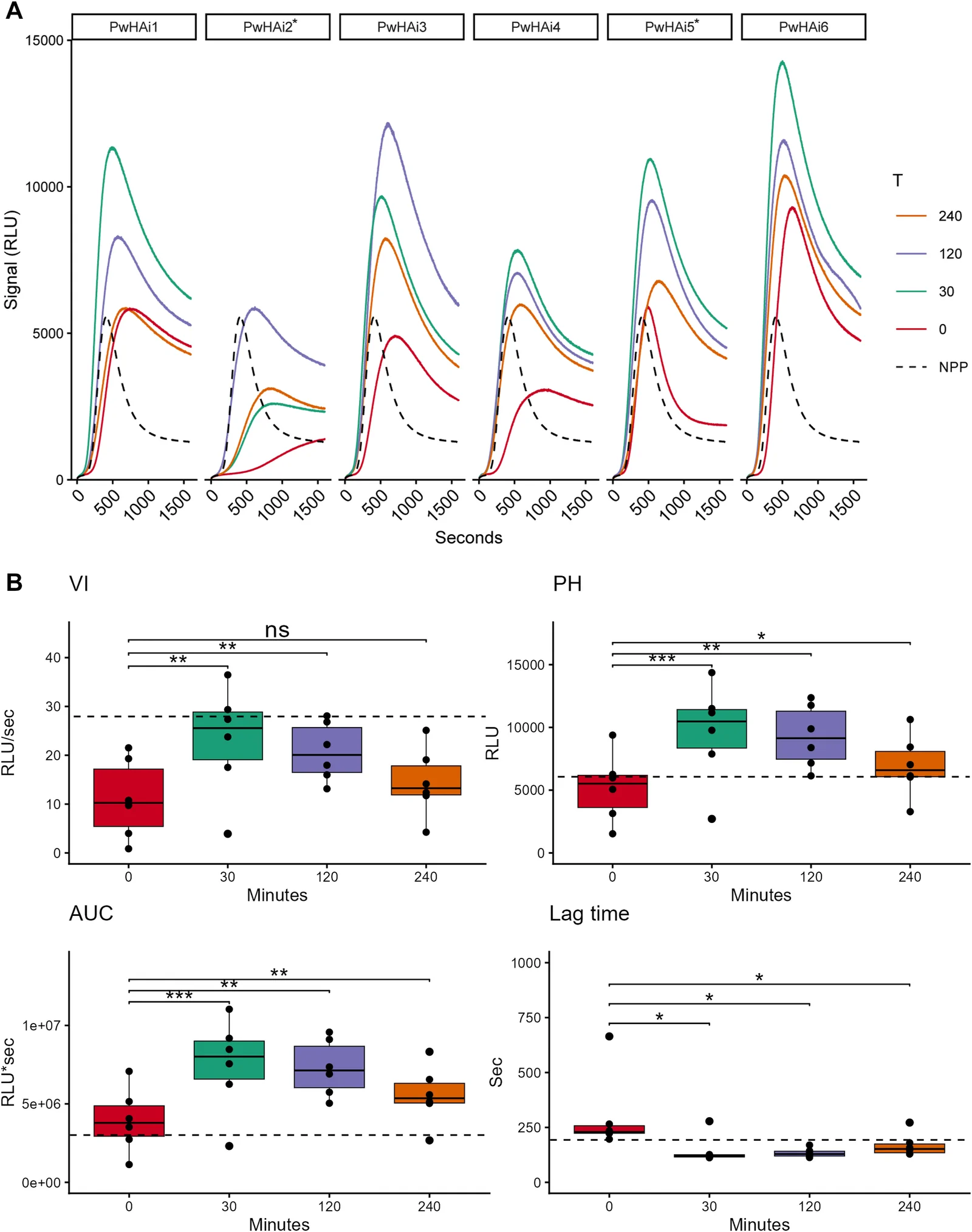
Microfluidic-TGA_lum_ curves (A) and parameters (B) of persons with hemophilia A with inhibitors before (T = 0) and after (T = 30, 120, 240 minutes) treatment with APCC. (A) Each line represents the mean thrombin generation curve of 2 assays. The dotted line represents the mean TGA curve of 10 repeated measurements in normal pooled plasma. Participants with hemophilia A with inhibitors who received emicizumab prophylaxis in addition to APCC (participants 2 and 5) are indicated by an asterisk. (B) Asterisks denote unadjusted *P* values (∗*P* < .05; ∗∗*P* < .01; ∗∗∗*P* < .001). The dotted line represents the mean value of the parameter in NPP (10 repeated measurements). Pairwise comparisons were performed using repeated-measures *t*-test for PH, AUC, and VI and Wilcoxon signed-rank test for lag time. After Holm correction, the pairwise comparisons for PH, AUC, and VI remained statistically significant. APCC, activated prothrombin complex concentrate; AUC, area under the curve; NBU, Nijmegen Bethesda units; NPP, normal pooled plasma; PwHAi, participant with hemophilia A with inhibitors; PH, peak height; RLU, relative light units; TGA_lum_, chemiluminescent thrombin generation assay; VI, velocity index.

The thrombin generation curves of the microfluidic-TGA_lum_ of participants with hemophilia A with inhibitors treated with APCC are shown in Figure 3A, and parameters are reported in Supplementary Table 1. Treatment with APCC increased thrombin generation in persons with hemophilia A with inhibitors as measured by the microfluidic-TGA_lum_, showing a maximal effect at 30 minutes postinfusion, returning to baseline after 4 hours. TGA results are expressed as a percentage of the result obtained in NPP. At baseline, VI in persons with hemophilia A with inhibitors was decreased compared with NPP (mean VI 40% ± 29%) and reached near-normal values 30 minutes after APCC treatment (mean VI 82% ± 39%). Lag time was prolonged at baseline (mean lag time 156% ± 93%) compared with NPP and normalized 30 minutes after treatment (mean lag time 75% ± 34%), with the effect persisting for >4 hours. Thrombin PH was considerably lower at baseline compared with NPP in participants 2 and 4, while reduced AUC values at baseline were observed exclusively in participant 2. Both thrombin PH and AUC increased following APCC treatment, reaching substantially higher than normal values in 5 participants with hemophilia A with inhibitors at 30 minutes postinfusion, with mean PH of 158% ± 66% and mean AUC of 248% ± 99% compared with NPP. After reaching peak levels 30 minutes after APCC treatment, PH and AUC dropped but did not return to baseline levels at 4 hours after treatment.

The effect of APCC treatment was statistically significant across all measured parameters (Figure 3B). VI, PH, and AUC increased significantly (repeated measures ANOVA, *P* < .001 for all), while lag time decreased (Friedman test, *P* = .002). The magnitude of the treatment effect varied considerably between persons with hemophilia A with inhibitors, with coefficients of variation for change per U/kg APCC dose 30 minutes posttreatment of 36%, 43%, 37%, and 93% for PH, AUC, VI, and lag time, respectively.

#### 3.2.3 TGA_Ceveron-RB_

**Figure 4:**
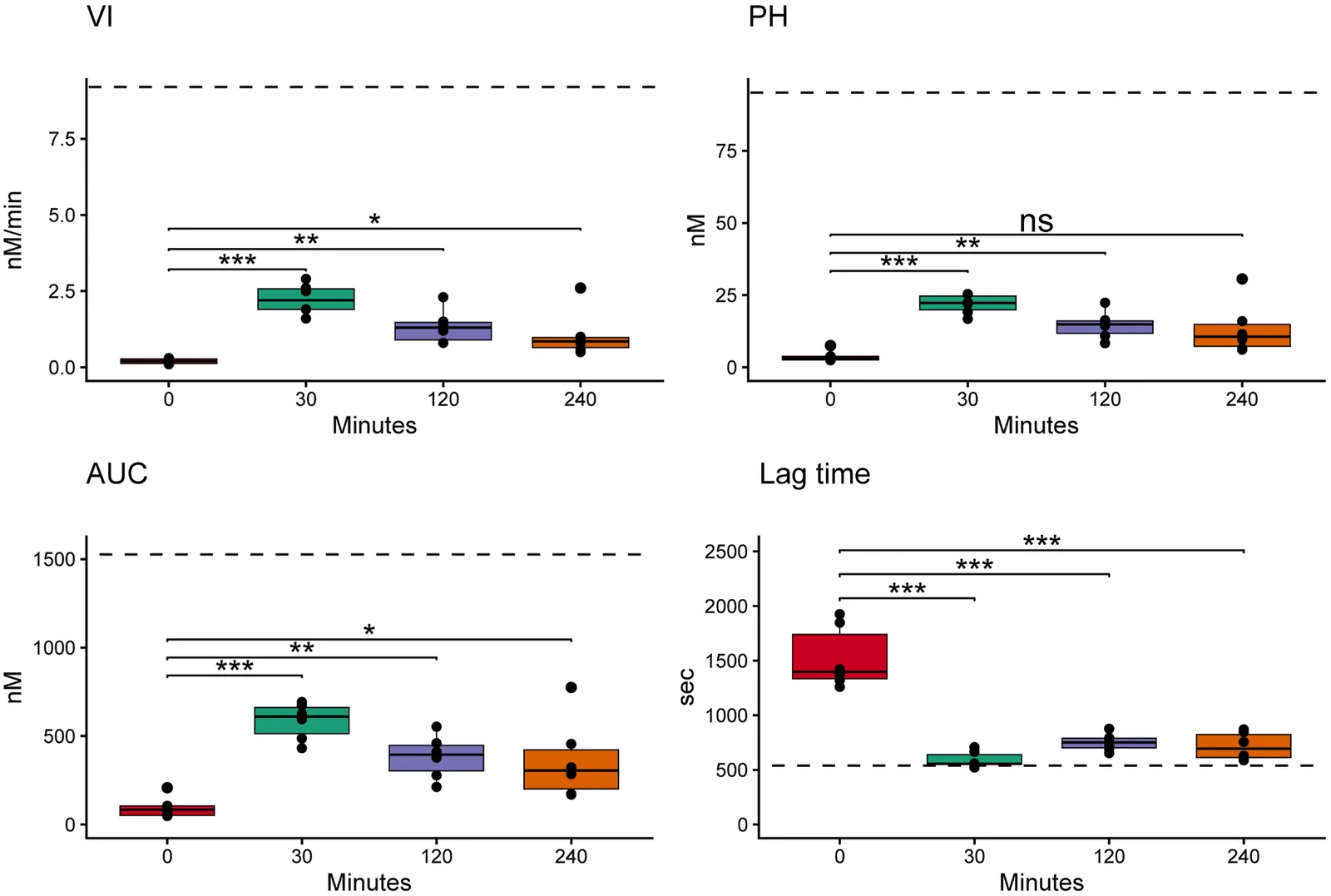
TGA_Ceveron-RB_ parameters in thawed samples before and after treatment with APCC. T: time in minutes before (0) or after (30, 120, 240) APCC treatment. Asterisks denote unadjusted *P* values (∗*P* < .05; ∗∗*P* < .01; ∗∗∗*P* < .001). The dotted line represents the mean value of the parameter in normal pooled plasma (2 repeated measurements in the same run). Pairwise comparisons were performed using repeated-measures *t*-test for all parameters. Significance was retained after Holm correction across all parameters. APCC, activated prothrombin complex concentrate; AUC, area under the curve; ns, not significant; PH, peak height; TGA_Ceveron-RB_, thrombin generation assay with Ceveron reagent B; VI, velocity index.

Similarly to the microfluidic-TGA_lum_, a significant effect of treatment with APCC on all TGA parameters was observed with the TGA_Ceveron-RB_, with a profile identical to that observed in the microfluidic-TGA_lum_: peak levels of VI, PH, and AUC after 30 minutes and a drop thereafter, but not returning to baseline levels at 4 hours after treatment (Figure 4, Supplementary Table 1). However, thrombin generation remained remarkably low in all samples compared with NPP. Only lag time approached normal values, reaching a mean of 109% (± 14%) relative to NPP 30 minutes after APCC administration.

#### 3.2.4 TGA_Ceveron-RCH_

**Figure 5:**
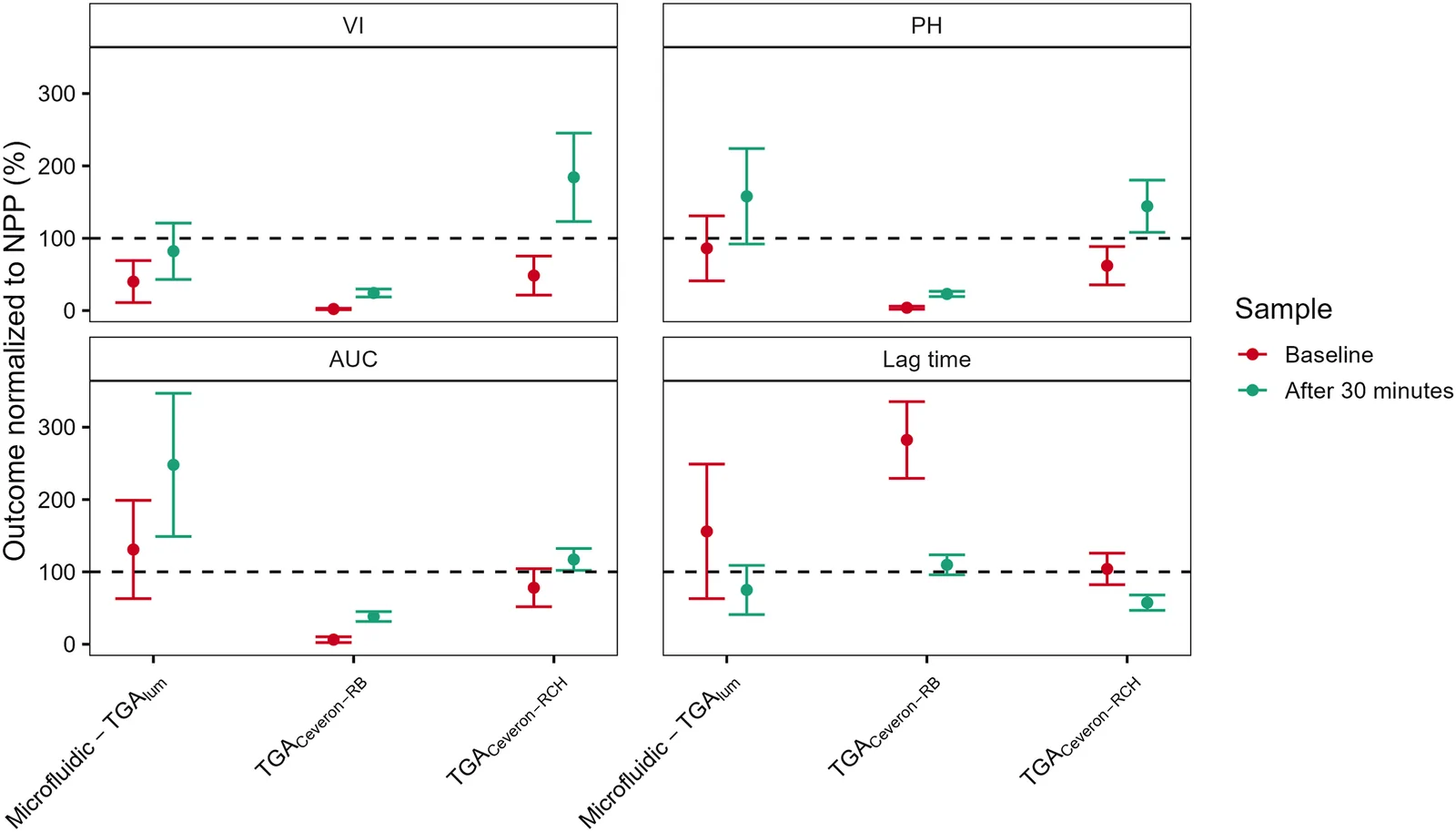
TGA outcome (mean ± standard deviation) of samples from participants with hemophilia A with inhibitors before and after treatment with activated prothrombin complex concentrate across TGA assays. AUC, area under the curve; NPP, normal pooled plasma; PH, peak height; TGA, thrombin generation assay; TGA_Ceveron-RB_, thrombin generation assay with Ceveron reagent B; TGA_Ceveron-RCH_, thrombin generation assay with Ceveron reagent C-high; TGA_lum_, chemiluminescent thrombin generation assay; VI, velocity index.

As thrombin generation in the TGA_Ceveron-RB_ was very low in all samples from participants with hemophilia A with inhibitors, the samples obtained at baseline and 30 minutes after treatment were retested with the TGA_Ceveron-RB_ and TGA_Ceveron-RCH_. In contrast to the persistent low thrombin generation observed in the TGA_Ceveron-RB_, thrombin generation reached normal or supranormal levels after APCC administration in the TGA_Ceveron-RCH_ (Figure 5, Supplementary Table 1). For this retesting, the samples had undergone prior thawing and refreezing. There was no evidence of significant influence of the thawing and refreezing on TGA results, with a mean difference in PH in TGA_Ceveron-RB_ results of −0.5 (± 4.5 nM) compared with samples analyzed before thawing and refreezing.

#### 3.2.5 Comparison of microfluidic-TGA_lum_ results with TGA_Ceveron-RB_ and TGA_Ceveron-RCH_ results measured in persons with hemophilia A with inhibitors treated with APCC

**Figure 6:**
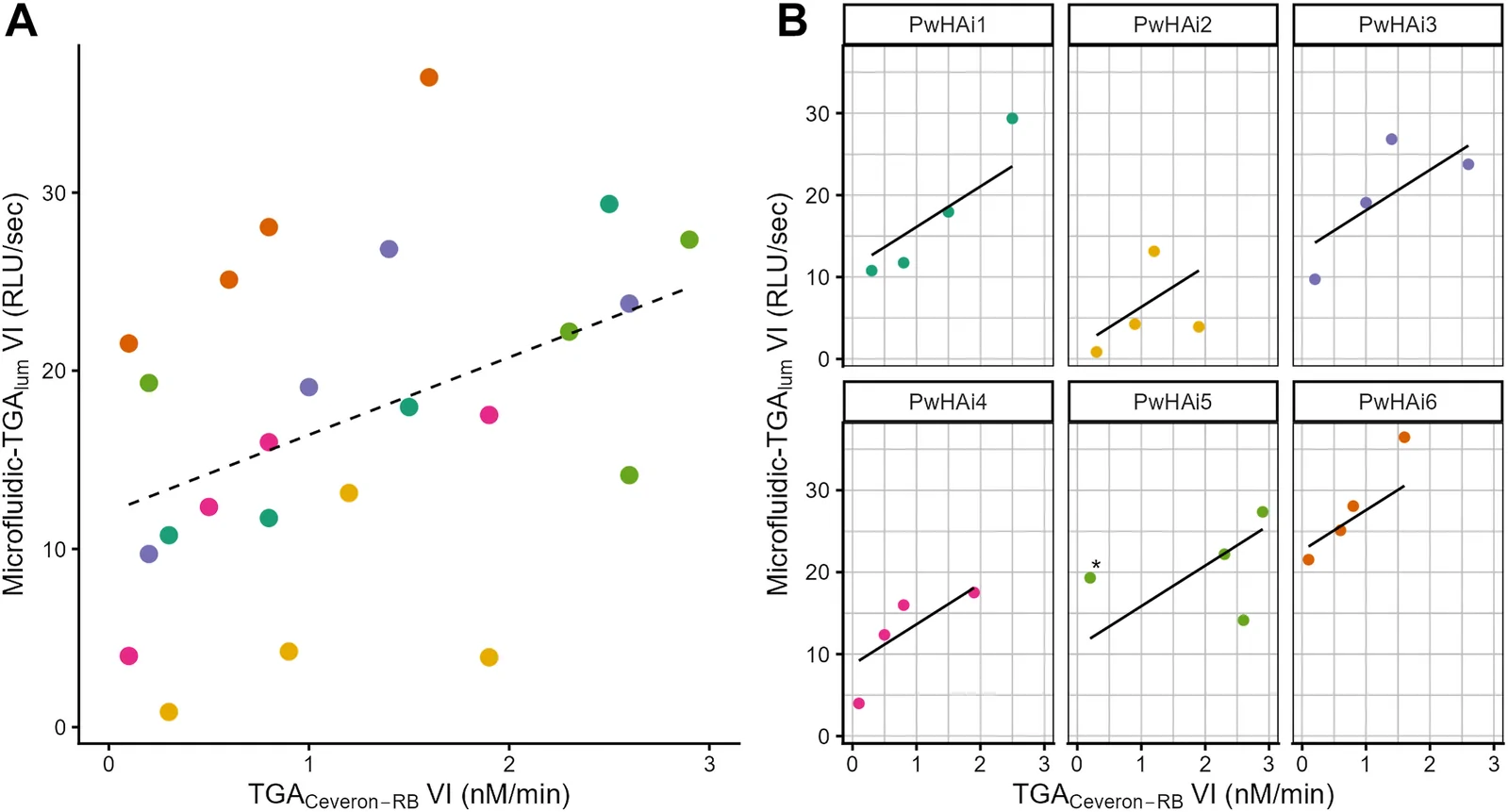
Comparison of TGA_Ceveron-RB_ VI and microfluidic-TGA_lum_ VI. (A) Correlation across all data points with linear regression line (dashed line). (B) Correlation within individual patients using multilevel regression lines with varying intercepts and fixed slopes (solid lines). ∗The T = 0 minutes sample of participant 5 was an influential case (Cook’s distance 1.41) in the model. PwHAi, participant with hemophilia A with inhibitors; TGA_Ceveron-RB_, thrombin generation assay with Ceveron reagent B; TGA_lum_, chemiluminescent thrombin generation assay; VI, velocity index.

Correlations between the microfluidic-TGA_lum_ and TGA_Ceveron-RB_ parameters were weak-to-moderate, with Pearson correlation coefficients for VI, PH, AUC, and lag time of 0.42, 0.29, 0.28, and 0.52, respectively (Figure 6A). *P* values, adjusted for multiple testing, were > .05 for all parameters. However, inspection of the data suggested that the poor correlation was primarily driven by between-subject differences (Figure 6B). To explore this, an unplanned multilevel regression was performed with random intercepts with and without random slopes. Compared with standard linear regression, estimating microfluidic-TGA_lum_ VI from TGA_Ceveron-RB_ VI, adding a random intercept for individual persons with hemophilia A with inhibitors significantly improved the model (χ^2^_1_ 12.2, *P* = < .001). The AIC decreased from 174.98 to 164.82, indicating a better model fit. In contrast, also allowing for varying slopes between persons with hemophilia A with inhibitors did not further improve the model (χ^2^_1_ 2.48, *P* = .29). In the random intercept model, 74% of the variation in the microfluidic-TGA_lum_ PH was accounted for by the TGA_Ceveron-RB_ VI and the donor-specific intercepts (conditional *R*^2^ [39]). This indicates that the weak correlation between the microfluidic-TGA_lum_ and TGA_Ceveron-RB_ was partially explained by differing baseline levels of VI, rather than by differing responses to APCC treatment across the assays.

Comparison of other parameters between the assays yielded similar results (Supplementary Table 2), except for lag time, where inclusion of random intercept did not significantly improve the model.

In contrast to the TGA_Ceveron-RB_ results, the TGA_Ceveron-RCH_ results of the baseline and 30 minutes posttreatment samples correlated more strongly with those of the microfluidic-TGA_lum_, with Pearson correlation coefficients of 0.76 (*P* = .004), 0.77 (*P* = .003), 0.75 (*P* = .005), and 0.75 (*P* = .005) for VI, PH, AUC, and lag time, respectively. These correlations remained significant after correction for multiple testing using the Holm method.

#### 3.2.6 Influence of prothrombin levels on thrombin generation

Repeated treatment with APCC results in accumulation vitamin K-dependent coagulation factors, the most abundant being prothrombin [11]. Elevated prothrombin levels, in turn, are associated with enhanced thrombin generation [[40], [41], [42], [43]], although it is unclear if this constitutes a significant role in the context of repeated treatment with APCC. Since the process of coagulation factor activation proceeds on phospholipids, the accumulation of prothrombin may hamper the rate of activation potentially in the TGA_Ceveron-RB_, which employs a low concentration of phospholipids. To better understand the differences in TGA results, both between persons with hemophilia A with inhibitors and between assay methodologies, the effect of prothrombin levels on TGA parameters was assessed. Prothrombin levels in samples obtained from patients were measured using a one-stage prothrombin assay using the ACL-TOP analyzer (Werfen) according to the instructions of the manufacturer. Additionally, NPP was spiked with human prothrombin (Sigma-Aldrich) and tested with the microfluidic-TGA_lum_, TGA_Ceveron-RB_, and TGA_Ceveron-RCH_.

Levels of prothrombin were measured in samples from participants with hemophilia A with inhibitors at baseline and 30 minutes after APCC treatment (Table). Baseline levels were elevated in all donors, except for participant 5, who did not receive APCC prophylaxis. The highest baseline prothrombin levels were observed in participant 1 (225%), who received his last prophylactic APCC dose one day before the study.

After APCC treatment, prothrombin levels increased in all donors, reaching levels between 287% and >600%. Baseline prothrombin levels did not correlate significantly with baseline microfluidic-TGA_lum_ or TGA_Ceveron-RB_ parameters nor with the intercepts of the multilevel regression models comparing the results of the microfluidic-TGA_lum_ and the TGA_Ceveron-RB_ (data not shown).

In the microfluidic-TGA_lum_ and to a lesser extent in the TGA_Ceveron-RCH_, thrombin generation increased in NPP spiked with incremental doses of prothrombin. In the TGA_Ceveron-RB_, thrombin generation also increased up to a spiked prothrombin concentration of 0.7 to 1.4 μM. However, further increases in prothrombin resulted in reduced PH in this assay, reaching 56% to 61% of the value observed in the nonspiked sample at 2.8 μM of spiked prothrombin (Supplementary Figure 2).

## 4 Discussion

### 4.1 Main findings

This study constitutes the first report on the use of the microfluidic-TGA_lum_ measured with the EnzySystem in clinical samples obtained from persons with hemophilia A with inhibitors treated with APCC, with or without emicizumab, who ultimately might benefit significantly from advanced monitoring approaches. Our results showed that the microfluidic-TGA_lum_ detects the increased hemostatic potential in plasma of APCC, both after spiking of APCC in FVIII-deficient plasma and in plasma from persons with hemophilia A with inhibitors treated with APCC, with notable heterogeneity in response between different samples from persons with hemophilia A with inhibitors. In samples spiked with APCC, a strong correlation of microfluidic-TGA_lum_ results with TGA_Ceveron-RB_ results was observed. However, in samples of persons with hemophilia A with inhibitors treated with APCC, the strength of the correlation was weak, in part due to subject-specific differences in TGA parameters across assays. Furthermore, the microfluidic-TGA_lum_ revealed hypercoagulable thrombin generation curves, whereas thrombin generation remained very low in the TGA_Ceveron-RB_. As the included persons with hemophilia A with inhibitors did not experience thrombotic or excessive bleeding events, it is unclear which of the evaluated TGA best reflects the clinical phenotype of persons with hemophilia A with inhibitors. Achieving this goal requires longitudinal studies in which TGA is performed at several moments in time, including at the time of bleeding or thrombotic complications.

### 4.2 Comparison with previous literature

The findings of our *in vitro* spiking experiments align with those of previous spiking studies, demonstrating a dose-dependent effect of APCC in FVIII-deficient plasma on PH and AUC, with normalization occurring at concentrations between 0.4 and 1 U/mL [11,29,[44], [45], [46], [47]]. The variability in TGA response between samples observed in the current study, was also reported by Luna-Záizar et al. [48], who observed variability in treatment response between samples of different persons with hemophilia A with inhibitors, owing in part to the kinetics of the inhibitor in each sample. Others confirmed augmented thrombin generation after treatment of persons with hemophilia A with inhibitors with APCC [11,29,30,49]. Ettingshausen et al. [49] monitored 9 prophylactic administrations of APCC in 5 children with hemophilia A with inhibitors. While AUC increased in all treatment episodes, the response was limited in 1 participant with a severe bleeding phenotype despite prophylaxis. This participant required twice-daily doses of APCC to prevent bleeding. Dargaud et al. [30] employed a 3-step TGA-based approach for the periinterventional treatment of 6 persons with hemophilia A with inhibitors. Here, TGA was used to determine the optimal bypassing agent and dose for individual persons with hemophilia A with inhibitors in spiked patient samples. Based on these results, perioperative treatment plans were created, with recombinant FVIIa selected for 3 patients and APCC for 3 patients. TGA was subsequently used to confirm efficacy after a trial treatment dose and to monitor treatment during surgery. Using this approach, large differences in efficacy of bypassing agents across persons with hemophilia A were observed. Clinical outcomes were optimal in all but 1 person with hemophilia A with inhibitors, in whom all the tested bypassing agents showed limited *in vitro* efficiency. Together, these studies provide early evidence confirming the clinical validity and implications of the TGA.

In the present study, TG curves were not elevated in the 2 participants with hemophilia A with inhibitors who received emicizumab in addition to APCC, compared with those who did not receive emicizumab. This observation contrasts with previous spiking studies that demonstrated striking hypercoagulable thrombin generation profiles when combining emicizumab even with very low levels (0.05 U/mL) of APCC [44,45,50,51]. However, spiking experiments do not account for the distribution and elimination of APCC components *in vivo*, which may differ between persons with hemophilia A with inhibitors and may add to the heterogeneity in treatment response. Our findings align more closely to those of Kizilocak et al. [52], who demonstrated a more limited synergistic effect of emicizumab and APCC when administered *in vivo*. In their study, which employed a dose-escalation approach guided by repeated TGAs, 6 of 9 children with hemophilia A with inhibitors were eligible for the highest dose of 75 U/kg bodyweight APCC, and none of the study participants experienced adverse treatment effects.

### 4.3 Discrepancy between microfluidic-TGA_lum_ and TGA_Ceveron-RB_ results

PH and especially AUC measured by the microfluidic-TGA_lum_ were remarkably elevated in the samples from patients treated with APCC, whereas thrombin generation remained very low in the TGA_Ceveron-RB_. Variability in preanalytical handling in clinical practice, including blood collection, centrifugation, transfer, and storage conditions, is a common source of differences between assay results [53]. However, in the present study, these procedures were identical across assays. Nonetheless, some degree of between-assay differences is to be expected, as TGA methodologies differ significantly with respect to reagent composition, thrombin substrate characteristics, and data analysis [32]. Consequently, the results of different TGA methodologies are not interchangeable, as already shown previously [38,54]. The assays included in this study differ in reagent composition, with the TGA_Ceveron-RB_ employing very low levels of phospholipids compared with the microfluidic-TGA_lum_, whereas the TGA_Ceveron-RCH_ contains high levels of phospholipids and tissue factor, although exact concentrations remain undisclosed [38]. Additionally, all commercially available TGAs employ a fluorescent readout principle, necessitating correction for the inner filter effect, whereas the chemiluminescent readout of the microfluidic-TGA_lum_ does not require such correction. Nevertheless, the extent of the observed discrepancies between assay results remains striking even when accounting for these methodological differences.

The cause of the differences between the microfluidic-TGA_lum_ and the TGA_Ceveron-RB_ appears to be present in the samples of the persons with hemophilia A with inhibitors treated with APCC, 5 of whom had received APCC for an extended period, as these aberrant findings were not observed in the spiking experiments. Because the high results were not observed in the spiking experiments, we can exclude cross-reactivity of APCC components with the thrombin substrate in the microfluidic-TGA_lum_ as a potential cause. Also, previous experiments showed no significant contribution of contact activation within the microfluidic system of the microfluidic-TGA_lum_ platform [35], thereby also excluding unintended intrinsic activation as an explanation. However, the differences in signals could hypothetically be explained by α_2_-macroglobulin-thrombin (α_2_M-thrombin) handling, accumulation of other coagulation factors, and phospholipid concentration.

α_2_M-thrombin could potentially contribute to the high PH and AUC observed in the microfluidic-TGA_lum_. Under normal *in vitro* conditions, antithrombin is available at sufficiently high concentrations (∼2.5 μM [55]) to inactivate the majority of formed thrombin, even if all prothrombin (∼1.4 μM [55]) is converted. However, when prothrombin levels are elevated and the total amount of *in vitro* thrombin formation is increased, the concentration of antithrombin may be insufficient, and a greater proportion of thrombin might be inhibited by α_2_M, as is also observed in antithrombin-deficient states [56,57]. While α_2_M-thrombin is biologically inactive, it can still cleave the chemiluminescent and fluorescent substrates used in TGAs [58]. The microfluidic-TGA_lum_ does not employ algorithms to account for the signal resulting from α_2_M-thrombin. Consequently, the thrombin generation curve of the microfluidic-TGA_lum_ does not return to the baseline at the end of the assay but instead shows a residual signal that is caused by α_2_M-thrombin [35]. As the α_2_M-thrombin effect primarily influences AUC [58,59], it could potentially partially explain the current findings. Supporting this role, the microfluidic-TGA_lum_ curves of donor samples showed an elevated tail and a slower decline in signal after the peak compared with NPP TGA curves, even when the PH was comparable (Figure 3A, participants 1 and 3). However, if α_2_M-thrombin plays a significant role in the microfluidic-TGA_lum_ results, a similar effect should have been observed in the TGA_Ceveron-RB_, as this assay also does not employ algorithms to account for α_2_M-thrombin. Therefore, we concluded that different mechanisms seem to impact the TGA_Ceveron-RB_, where thrombin generation was extremely low across all patient samples, even after treatment with APCC.

Potentially, the very low concentration of phospholipids used in the TGA_Ceveron-RB_ may become limiting in the context of accumulation of coagulation factors due to repeated APCC administration. The concentrations of several coagulation factors, including prothrombin (*t*_1/2_ 60-70 hours) and FX (*t*_1/2_ 24-48 hours), gradually increase during prophylactic treatment with APCC, reaching a steady state after 3 months [11]. Increased levels of prothrombin enhance thrombin generation in cell-based models [42], in *in vitro* experiments [60], and in patient samples [40,41,61]. In the present study, we confirmed the presence of high prothrombin levels in persons with hemophilia A with inhibitors receiving APCC prophylaxis, as well as an increasing effect of elevated prothrombin concentrations on thrombin generation in the microfluidic-TGA_lum_. In contrast, a decrease in thrombin generation was observed in the TGA_Ceveron-RB_ in NPP spiked with high concentrations of prothrombin (Supplementary Figure 2). The low phospholipid concentration used in this assay may increase its sensitivity to altered coagulation factor ratios. One possible explanation is that phospholipid binding spaces might be occupied by factors in relative abundance, such as prothrombin, potentially limiting the assembly of the tissue factor and prothrombinase complex. Supporting this hypothesis, we did not observe a prothrombin-induced decrease in thrombin generation in the TGA_Ceveron-RCH_, which utilizes higher levels of phospholipids. Normalization of TGA parameters after APCC treatment was observed with this assay, aligning more closely the findings of previous studies and the microfluidic-TGA_lum_ results. However, it remains unclear whether this effect is due to the higher concentration of phospholipids in the assay, as it also utilizes a higher concentration of tissue factor. In the context of high tissue factor levels, coagulation is less dependent on the formation of the FVIII/FIXa/FX tenase complex on the phospholipid surface. For this reason, TGA with high tissue factor concentrations is considered unsuitable for monitoring hemophilia [62].

The differences observed between the TGA methods emphasize the importance of TGA standardization. Unfortunately, the issue of TGA standardization has not been fully resolved [63], despite the publication of international recommendations [53] and the transition from manual to (semi-)automated methodologies [32]. While normalization of assay results to externally provided reference plasma could improve the comparability of results obtained at different sites [31], data aggregation will remain difficult if assay methodologies differ between sites, reagents vary between and even within platforms, and assay-specific differential sensitivities to specific factors—as found in this study—are not further elucidated.

### 4.4 Strengths and limitations

The strengths of this study include sequential monitoring of APCC after administration, along with the comparison with other TGA platforms. This approach provides meaningful insights to support the further development of the EnzySystem and increase the understanding of the complexities involved with monitoring APCC treatment.

However, some limitations must be acknowledged. First, as this was a strictly observational study, no washout period was incorporated in the study. This increased the heterogeneity of the participants, which was already substantial due to differences in treatment regimens and comorbidities. Additionally, the sample size was small, and no long-term follow-up period was included. Therefore, the study does not allow for correlation of assay results with meaningful clinical outcomes. Finally, several analyses were driven by the observation of unexpected results. Although deviations from the initial analytical plan are highlighted in the manuscript, the inherent limitations of post hoc analyses should be acknowledged. Lastly, caution is warranted in drawing conclusions regarding optimal TGA monitoring based on the current data, as we only evaluated the results of the microfluidic-TGA_lum_ against 2 other TGA techniques, both based on the Ceveron platform. Other Ceveron assay kits and TGA platforms (eg, the St Genesia and Calibrated Automated Thrombogram) were not included in this first field study of the EnzySystem. Additionally, enabling optimal selection of bypassing agents based on TGA monitoring will require evaluation across all available bypassing agents, which was not performed in the present study. Evaluation of these additional techniques and treatment conditions will be necessary to support more comprehensive and well-informed conclusions.

## 5 Conclusion and Future Recommendations

Our findings provide initial evidence for the applicability of the microfluidic-TGA_lum_ to monitor persons with hemophilia A with inhibitors treated with APCC either alone or in combination with emicizumab. However, further validation is required, especially for monitoring combined treatment with APCC and emicizumab, given the risks of thrombosis associated with such therapy. The results also highlight challenges with performing TGA in complex patient samples, which may be influenced not only by the monitored drug, but also by variations in coagulation factor activity levels, different kinetic effects of the inhibitors, sample conditions, comedications, comorbidities, and assay methodology. Comprehending the limitations and sensitivities of available TGAs to these factors is essential for safe clinical use, and targeted *in vitro* studies evaluating all available TGA platforms are required to improve this knowledge. Ultimately, prospective studies that correlate clinical events with TGA results are needed to better understand the potential benefit of TGA. The heterogeneity in patient characteristics, combined with the relative rarity of hemophilia A and the low annual bleeding rate with modern treatment options, underscores the importance of standardizing TGA methodologies and data management. Such standardization will facilitate the aggregation of data across studies, which will ultimately be pivotal to deriving meaningful and reliable conclusions regarding the clinical use of TGA.

## Figures and Tables

**Supplementary Figure S2:**