# Supplementary material for: A miniaturized chemiluminescent thrombin generation assay to assess the effect of APCC in persons with hemophilia A with inhibitors: an observational study
Source: Res Pract Thromb Haemost. 2026 Aug 27;10(6):106920. doi: 10.1016/j.rpth.2026.106920 (PMC13597294; doi:10.1016/j.rpth.2026.106920)
Supplement: Supplementary file 1 [file mmc1.docx]

**Supplemental Table 1** TGA outcomes normalized to normal pooled plasma

**Microfluidic-TGA_lum­_**

| **PwHA** | **Time after aPCC treatment (min)** | **Peak height** | **Lag time** | **Area under curve** | **Velocity index** |
| --- | --- | --- | --- | --- | --- |
| PwHA1 | Baseline | 99 | 121 | 171 | 39 |
| PwHA2 | Baseline | 25 | 344 | 38 | 3 |
| PwHA3 | Baseline | 84 | 116 | 135 | 35 |
| PwHA4 | Baseline | 52 | 137 | 91 | 14 |
| PwHA5 | Baseline | 103 | 102 | 117 | 69 |
| PwHA6 | Baseline | 155 | 116 | 235 | 77 |
| PwHA1 | 30 | 190 | 58 | 305 | 105 |
| PwHA2 | 30 | 45 | 144 | 77 | 14 |
| PwHA3 | 30 | 161 | 64 | 251 | 85 |
| PwHA4 | 30 | 130 | 65 | 207 | 63 |
| PwHA5 | 30 | 184 | 61 | 281 | 98 |
| PwHA6 | 30 | 237 | 59 | 366 | 131 |
| PwHA1 | 120 | 138 | 74 | 229 | 64 |
| PwHA2 | 120 | 101 | 88 | 167 | 47 |
| PwHA3 | 120 | 204 | 71 | 318 | 96 |
| PwHA4 | 120 | 118 | 62 | 191 | 57 |
| PwHA5 | 120 | 163 | 62 | 244 | 79 |
| PwHA6 | 120 | 194 | 59 | 302 | 100 |
| PwHA1 | 240 | 100 | 93 | 170 | 42 |
| PwHA2 | 240 | 54 | 141 | 89 | 15 |
| PwHA3 | 240 | 139 | 76 | 217 | 68 |
| PwHA4 | 240 | 101 | 68 | 167 | 44 |
| PwHA5 | 240 | 116 | 82 | 185 | 51 |
| PwHA6 | 240 | 175 | 67 | 276 | 90 |

**TGA_Ceveron-RB_**

| **PwHA** | **Time after aPCC treatment (min)** | **Peak height** | **Lag time** | **Area under curve** | **Velocity index** |
| --- | --- | --- | --- | --- | --- |
| PwHA1 | Baseline | 4 | 254 | 7 | 3 |
| PwHA2 | Baseline | 8 | 263 | 14 | 3 |
| PwHA3 | Baseline | 4 | 233 | 7 | 2 |
| PwHA4 | Baseline | 3 | 342 | 3 | 1 |
| PwHA5 | Baseline | 3 | 357 | 3 | 2 |
| PwHA6 | Baseline | 3 | 244 | 4 | 1 |
| PwHA1 | 30 | 27 | 102 | 45 | 27 |
| PwHA2 | 30 | 23 | 131 | 44 | 21 |
| PwHA3 | 30 | 27 | 97 | 41 | 28 |
| PwHA4 | 30 | 20 | 123 | 32 | 21 |
| PwHA5 | 30 | 24 | 102 | 39 | 32 |
| PwHA6 | 30 | 18 | 103 | 28 | 17 |
| PwHA1 | 120 | 15 | 133 | 25 | 16 |
| PwHA2 | 120 | 17 | 162 | 30 | 13 |
| PwHA3 | 120 | 16 | 121 | 27 | 15 |
| PwHA4 | 120 | 11 | 147 | 18 | 9 |
| PwHA5 | 120 | 24 | 129 | 36 | 25 |
| PwHA6 | 120 | 9 | 144 | 14 | 9 |
| PwHA1 | 240 | 10 | 117 | 19 | 9 |
| PwHA2 | 240 | 17 | 161 | 30 | 10 |
| PwHA3 | 240 | 12 | 109 | 21 | 11 |
| PwHA4 | 240 | 6 | 157 | 11 | 5 |
| PwHA5 | 240 | 32 | 112 | 51 | 28 |
| PwHA6 | 240 | 7 | 140 | 11 | 7 |

**TGA_Ceveron-RCH_**

| **PwHA** | **Time after aPCC treatment (min)** | **Peak height** | **Lag time** | **Area under curve** | **Velocity index** |
| --- | --- | --- | --- | --- | --- |
| PwHA1 | Baseline | 83 | 89 | 93 | 78 |
| PwHA2 | Baseline | 38 | 133 | 56 | 23 |
| PwHA3 | Baseline | 95 | 79 | 111 | 78 |
| PwHA4 | Baseline | 45 | 113 | 67 | 29 |
| PwHA5 | Baseline | 33 | 123 | 44 | 22 |
| PwHA6 | Baseline | 77 | 89 | 98 | 60 |
| PwHA1 | 30 | 178 | 44 | 132 | 239 |
| PwHA2 | 30 | 96 | 74 | 96 | 102 |
| PwHA3 | 30 | 183 | 49 | 136 | 240 |
| PwHA4 | 30 | 117 | 64 | 111 | 133 |
| PwHA5 | 30 | 126 | 54 | 108 | 158 |
| PwHA6 | 30 | 166 | 59 | 120 | 235 |
