# Supplementary material for: A miniaturized chemiluminescent thrombin generation assay to assess the effect of APCC in persons with hemophilia A with inhibitors: an observational study
Source: Res Pract Thromb Haemost. 2026 Aug 27;10(6):106920. doi: 10.1016/j.rpth.2026.106920 (PMC13597294; doi:10.1016/j.rpth.2026.106920)
Supplement: Supplementary file 2 [file mmc2.docx]

**Supplemental table 2** Multilevel regression comparison of microfluidic-TGA_lum_ and TGA_Ceveron-RB_ results

| **Parameter** | | **Intercept (± SE)** | **Slope (± SE)** | **Random intercept SD** | **R^2^**  **R^2^_m_/R^2^_c_ ^a^** | **Model comparison (χ^2^(df), p)** |
| --- | --- | --- | --- | --- | --- | --- |
| VI | |  |  |  |  |  |
|  | Linear model | 12.05 (± 2.97) | 4.35 (± 1.99) | NA | 0.18 |  |
|  | Mixed model (random intercept) | 11.34 (± 3.52) | 4.94 (±1.30) | 5.00 | 0.20 / 0.74 | 12.2 (1), <0.001 |
| PH | |  |  |  |  |  |
|  | Linear model | 6199.6 | 115.8 | NA | 0.09 |  |
|  | Mixed model (random intercept) | 5295.2 (± 1409.4) | 183.3 (± 49.4) | 2912 | 0.17 / 0.77 | 13.8 (1), < 0.001 |
| AUC | |  |  |  |  |  |
|  | Linear model | 4,979*10^3^ (± 983*10^3^) | 3.0*10^3^ (± 2.4*10^3^) | NA | 0.07 |  |
|  | Mixed model (random intercept) | 3,957*10^3^ (± 1,107*10^3^) | 5.9*10^3^ (± 1.4 *10^3^) | 2,332*10^3^ | 0.19 / 0.80 | 15.0 (1), <0.001 |
| Lag time | |  |  |  |  |  |
|  | Linear model | 54.4 (± 50.7) | 0.15 (± 0.05) | NA | 0.27 |  |
|  | Mixed model (random intercept) | 58.0 (± 48.8) | 0.14 (± 0.04) | 62.8 | 0.25 / 0.53 | 3.1 (1), 0.08b |

*R^2^_c_ Conditional R^2^, R^2^_m_ Marginal R^2^*

*a: Marginal R^2^ indicates the proportion of the variance explained by the mixed model without incorporating random intercepts, whereas conditional R^2^ indicates the variance explained in the model including the random intercepts. Because variance is evaluated differently in linear and mixed models, marginal R² values are not directly comparable to R^2^ values from linear regression. b: Diagnostic plots of the model showed evidence of non-linearity, non-normality of the residuals, and heteroscedasticity, hampering the reliability of the model’s inferences*
